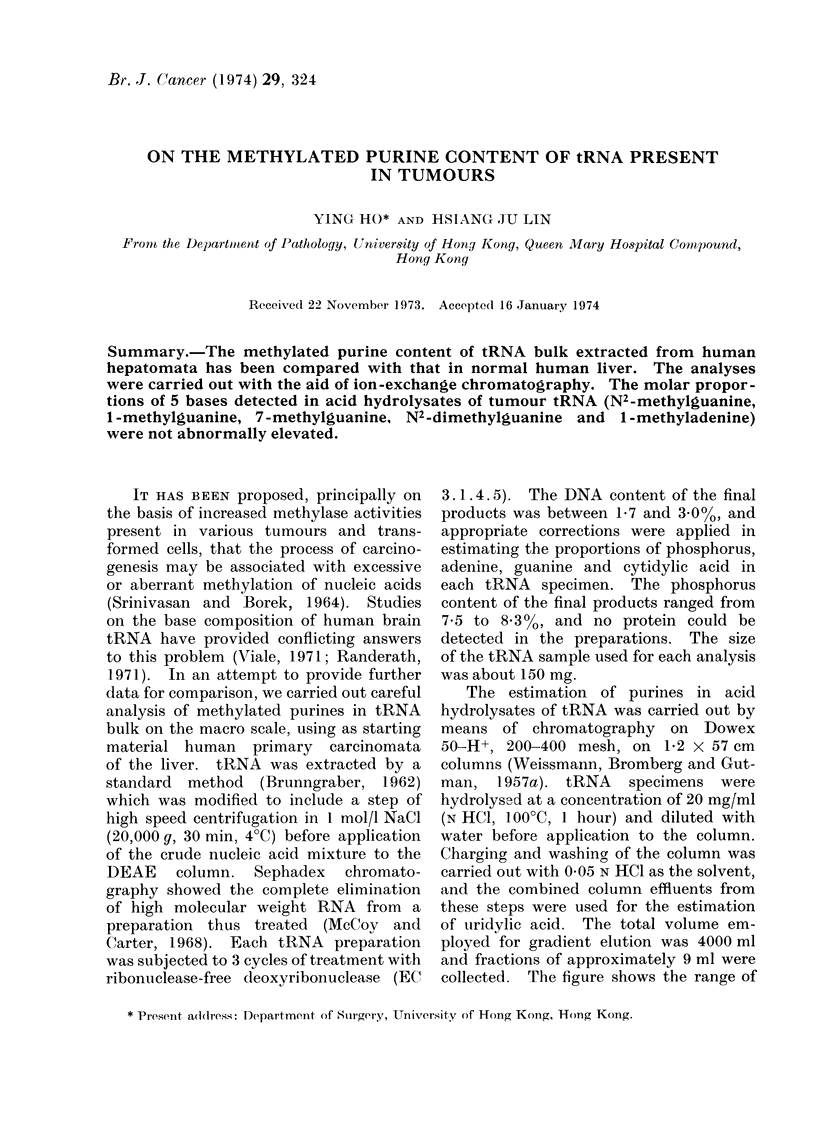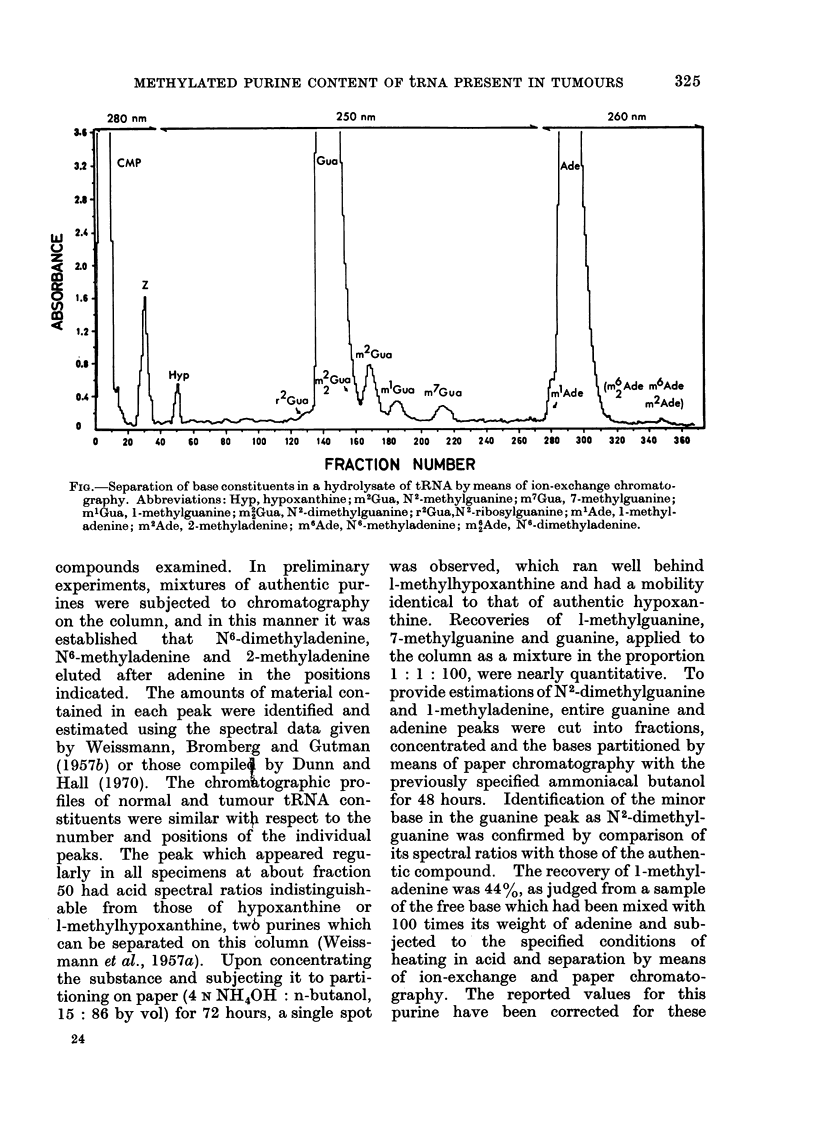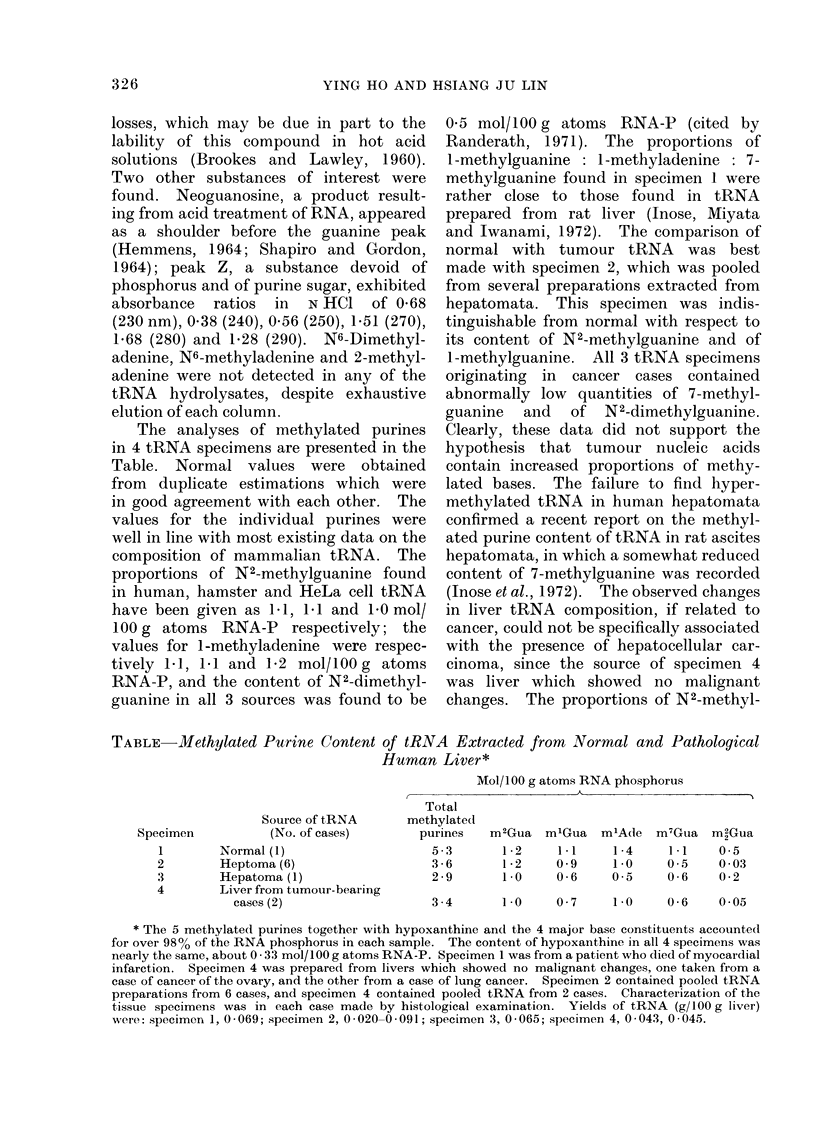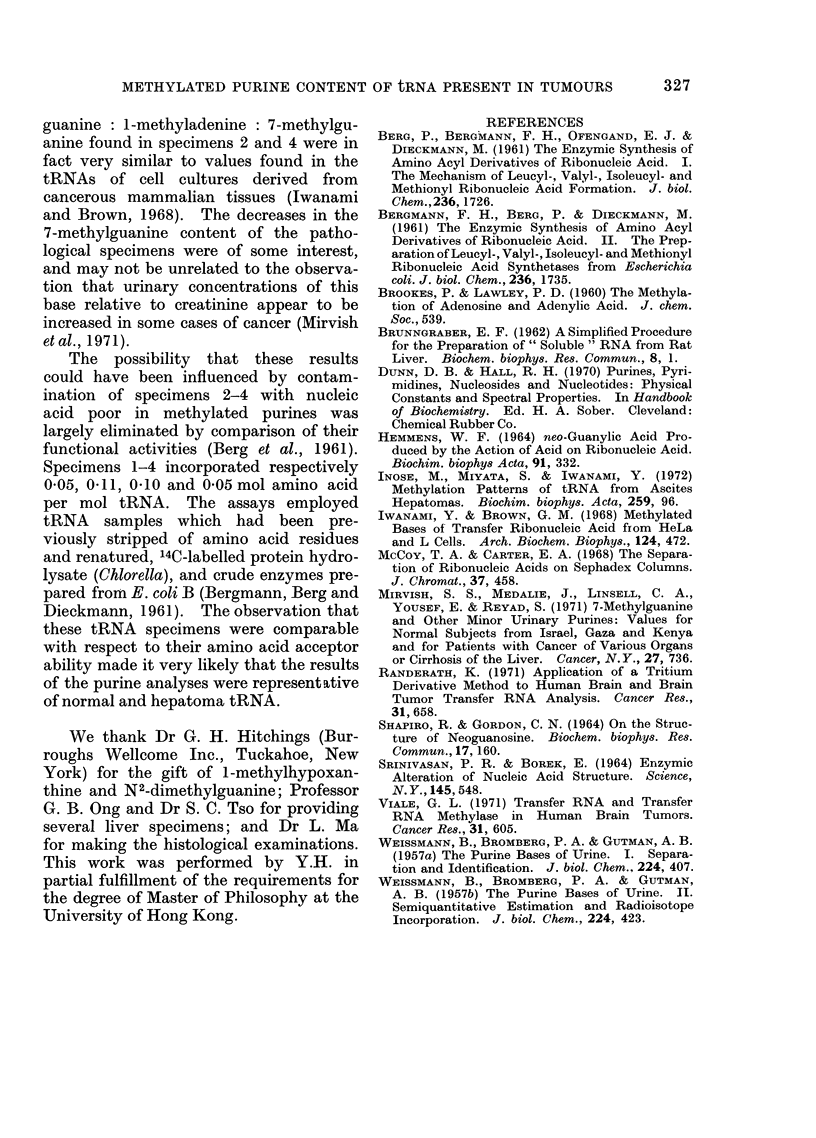# On the Methylated Purine Content of tRNA Present in Tumours

**DOI:** 10.1038/bjc.1974.74

**Published:** 1974-04

**Authors:** Ying Ho, Hsiang Ju Lin

## Abstract

The methylated purine content of tRNA bulk extracted from human hepatomata has been compared with that in normal human liver. The analyses were carried out with the aid of ion-exchange chromatography. The molar proportions of 5 bases detected in acid hydrolysates of tumour tRNA (N^2^-methylguanine, 1-methylguanine, 7-methylguanine, N^2^-dimethylguanine and 1-methyladenine) were not abnormally elevated.


*Br. J. Cancer* (1974) **29**, 324

### ON THE METHYLATED PURINE CONTENT OF tRNA PRESENT IN TUMOURS

YING HO* AND HSIANG JU LIN

*From the Department of Pathology, University of Hong Kong, Queen Mary Hospital Compound, Hong Kong*

Received 22 November 1973. Accepted 16 January 1974

**Summary.—The methylated purine content of tRNA bulk extracted from human hepatomata has been compared with that in normal human liver. The analyses were carried out with the aid of ion-exchange chromatography. The molar proportions of 5 bases detected in acid hydrolysates of tumour tRNA ($N^2$-methylguanine, 1-methylguanine, 7-methylguanine, $N^2$-dimethylguanine and 1-methyladenine) were not abnormally elevated.**

IT HAS BEEN proposed, principally on the basis of increased methylase activities present in various tumours and transformed cells, that the process of carcinogenesis may be associated with excessive or aberrant methylation of nucleic acids (Srinivasan and Borek, 1964). Studies on the base composition of human brain tRNA have provided conflicting answers to this problem (Viale, 1971; Randerath, 1971). In an attempt to provide further data for comparison, we carried out careful analysis of methylated purines in tRNA bulk on the macro scale, using as starting material human primary carcinomata of the liver. tRNA was extracted by a standard method (Brunngraber, 1962) which was modified to include a step of high speed centrifugation in 1 mol/l NaCl (20,000 *g*, 30 min, 4°C) before application of the crude nucleic acid mixture to the DEAE column. Sephadex chromatography showed the complete elimination of high molecular weight RNA from a preparation thus treated (McCoy and Carter, 1968). Each tRNA preparation was subjected to 3 cycles of treatment with ribonuclease-free deoxyribonuclease (EC 3.1.4.5). The DNA content of the final products was between 1·7 and 3·0%, and appropriate corrections were applied in estimating the proportions of phosphorus, adenine, guanine and cytidylic acid in each tRNA specimen. The phosphorus content of the final products ranged from 7·5 to 8·3%, and no protein could be detected in the preparations. The size of the tRNA sample used for each analysis was about 150 mg.

The estimation of purines in acid hydrolysates of tRNA was carried out by means of chromatography on Dowex 50–$H^+$, 200–400 mesh, on 1·2 × 57 cm columns (Weissmann, Bromberg and Gutman, 1957*a*). tRNA specimens were hydrolysed at a concentration of 20 mg/ml (N HCl, 100°C, 1 hour) and diluted with water before application to the column. Charging and washing of the column was carried out with 0·05 N HCl as the solvent, and the combined column effluents from these steps were used for the estimation of uridylic acid. The total volume employed for gradient elution was 4000 ml and fractions of approximately 9 ml were collected. The figure shows the range of

* Present address: Department of Surgery, University of Hong Kong, Hong Kong.

FIG.—Separation of base constituents in a hydrolysate of tRNA by means of ion-exchange chromatography. Abbreviations: Hyp, hypoxanthine; $m^2$Gua, $N^2$-methylguanine; $m^7$Gua, 7-methylguanine; $m^1$Gua, 1-methylguanine; $m^2_2$Gua, $N^2$-dimethylguanine; $r^2$Gua, $N^2$-ribosylguanine; $m^1$Ade, 1-methyladenine; $m^2$Ade, 2-methyladenine; $m^6$Ade, $N^6$-methyladenine; $m^6_2$Ade, $N^6$-dimethyladenine.

compounds examined. In preliminary experiments, mixtures of authentic purines were subjected to chromatography on the column, and in this manner it was established that $N^6$-dimethyladenine, $N^6$-methyladenine and 2-methyladenine eluted after adenine in the positions indicated. The amounts of material contained in each peak were identified and estimated using the spectral data given by Weissmann, Bromberg and Gutman (1957*b*) or those compiled by Dunn and Hall (1970). The chromatographic profiles of normal and tumour tRNA constituents were similar with respect to the number and positions of the individual peaks. The peak which appeared regularly in all specimens at about fraction 50 had acid spectral ratios indistinguishable from those of hypoxanthine or 1-methylhypoxanthine, two purines which can be separated on this column (Weissmann *et al.*, 1957*a*). Upon concentrating the substance and subjecting it to partitioning on paper (4 N $NH_4OH$ : n-butanol, 15 : 86 by vol) for 72 hours, a single spot was observed, which ran well behind 1-methylhypoxanthine and had a mobility identical to that of authentic hypoxanthine. Recoveries of 1-methylguanine, 7-methylguanine and guanine, applied to the column as a mixture in the proportion 1 : 1 : 100, were nearly quantitative. To provide estimations of $N^2$-dimethylguanine and 1-methyladenine, entire guanine and adenine peaks were cut into fractions, concentrated and the bases partitioned by means of paper chromatography with the previously specified ammoniacal butanol for 48 hours. Identification of the minor base in the guanine peak as $N^2$-dimethylguanine was confirmed by comparison of its spectral ratios with those of the authentic compound. The recovery of 1-methyladenine was 44%, as judged from a sample of the free base which had been mixed with 100 times its weight of adenine and subjected to the specified conditions of heating in acid and separation by means of ion-exchange and paper chromatography. The reported values for this purine have been corrected for these losses, which may be due in part to the lability of this compound in hot acid solutions (Brookes and Lawley, 1960). Two other substances of interest were found. Neoguanosine, a product resulting from acid treatment of RNA, appeared as a shoulder before the guanine peak (Hemmens, 1964; Shapiro and Gordon, 1964); peak Z, a substance devoid of phosphorus and of purine sugar, exhibited absorbance ratios in N HCl of 0·68 (230 nm), 0·38 (240), 0·56 (250), 1·51 (270), 1·68 (280) and 1·28 (290). $N^6$-Dimethyladenine, $N^6$-methyladenine and 2-methyladenine were not detected in any of the tRNA hydrolysates, despite exhaustive elution of each column.

The analyses of methylated purines in 4 tRNA specimens are presented in the Table. Normal values were obtained from duplicate estimations which were in good agreement with each other. The values for the individual purines were well in line with most existing data on the composition of mammalian tRNA. The proportions of $N^2$-methylguanine found in human, hamster and HeLa cell tRNA have been given as 1·1, 1·1 and 1·0 mol/100 g atoms RNA-P respectively; the values for 1-methyladenine were respectively 1·1, 1·1 and 1·2 mol/100 g atoms RNA-P, and the content of $N^2$-dimethylguanine in all 3 sources was found to be 0·5 mol/100 g atoms RNA-P (cited by Randerath, 1971). The proportions of 1-methylguanine : 1-methyladenine : 7-methylguanine found in specimen 1 were rather close to those found in tRNA prepared from rat liver (Inose, Miyata and Iwanami, 1972). The comparison of normal with tumour tRNA was best made with specimen 2, which was pooled from several preparations extracted from hepatomata. This specimen was indistinguishable from normal with respect to its content of $N^2$-methylguanine and of 1-methylguanine. All 3 tRNA specimens originating in cancer cases contained abnormally low quantities of 7-methylguanine and of $N^2$-dimethylguanine. Clearly, these data did not support the hypothesis that tumour nucleic acids contain increased proportions of methylated bases. The failure to find hypermethylated tRNA in human hepatomata confirmed a recent report on the methylated purine content of tRNA in rat ascites hepatomata, in which a somewhat reduced content of 7-methylguanine was recorded (Inose *et al.*, 1972). The observed changes in liver tRNA composition, if related to cancer, could not be specifically associated with the presence of hepatocellular carcinoma, since the source of specimen 4 was liver which showed no malignant changes. The proportions of $N^2$-methyl-

TABLE—*Methylated Purine Content of tRNA Extracted from Normal and Pathological Human Liver**

| Specimen | Source of tRNA (No. of cases) | Mol/100 g atoms RNA phosphorus: Total methylated purines | $m^2Gua$ | $m^1Gua$ | $m^1Ade$ | $m^7Gua$ | $m^2_2Gua$ |
|---|---|---|---|---|---|---|---|
| 1 | Normal (1) | 5·3 | 1·2 | 1·1 | 1·4 | 1·1 | 0·5 |
| 2 | Heptoma (6) | 3·6 | 1·2 | 0·9 | 1·0 | 0·5 | 0·03 |
| 3 | Hepatoma (1) | 2·9 | 1·0 | 0·6 | 0·5 | 0·6 | 0·2 |
| 4 | Liver from tumour-bearing cases (2) | 3·4 | 1·0 | 0·7 | 1·0 | 0·6 | 0·05 |

* The 5 methylated purines together with hypoxanthine and the 4 major base constituents accounted for over 98% of the RNA phosphorus in each sample. The content of hypoxanthine in all 4 specimens was nearly the same, about 0·33 mol/100 g atoms RNA-P. Specimen 1 was from a patient who died of myocardial infarction. Specimen 4 was prepared from livers which showed no malignant changes, one taken from a case of cancer of the ovary, and the other from a case of lung cancer. Specimen 2 contained pooled tRNA preparations from 6 cases, and specimen 4 contained pooled tRNA from 2 cases. Characterization of the tissue specimens was in each case made by histological examination. Yields of tRNA (g/100 g liver) were: specimen 1, 0·069; specimen 2, 0·020–0·091; specimen 3, 0·065; specimen 4, 0·043, 0·045.

guanine : 1-methyladenine : 7-methylguanine found in specimens 2 and 4 were in fact very similar to values found in the tRNAs of cell cultures derived from cancerous mammalian tissues (Iwanami and Brown, 1968). The decreases in the 7-methylguanine content of the pathological specimens were of some interest, and may not be unrelated to the observation that urinary concentrations of this base relative to creatinine appear to be increased in some cases of cancer (Mirvish *et al.*, 1971).

The possibility that these results could have been influenced by contamination of specimens 2–4 with nucleic acid poor in methylated purines was largely eliminated by comparison of their functional activities (Berg *et al.*, 1961). Specimens 1–4 incorporated respectively 0·05, 0·11, 0·10 and 0·05 mol amino acid per mol tRNA. The assays employed tRNA samples which had been previously stripped of amino acid residues and renatured, $^{14}$C-labelled protein hydrolysate (*Chlorella*), and crude enzymes prepared from *E. coli* B (Bergmann, Berg and Dieckmann, 1961). The observation that these tRNA specimens were comparable with respect to their amino acid acceptor ability made it very likely that the results of the purine analyses were representative of normal and hepatoma tRNA.

We thank Dr G. H. Hitchings (Burroughs Wellcome Inc., Tuckahoe, New York) for the gift of 1-methylhypoxanthine and N$^2$-dimethylguanine; Professor G. B. Ong and Dr S. C. Tso for providing several liver specimens; and Dr L. Ma for making the histological examinations. This work was performed by Y.H. in partial fulfillment of the requirements for the degree of Master of Philosophy at the University of Hong Kong.

REFERENCES

Berg, P., Bergmann, F. H., Ofengand, E. J. & Dieckmann, M. (1961) The Enzymic Synthesis of Amino Acyl Derivatives of Ribonucleic Acid. I. The Mechanism of Leucyl-, Valyl-, Isoleucyl- and Methionyl Ribonucleic Acid Formation. *J. biol. Chem.*, **236**, 1726.

Bergmann, F. H., Berg, P. & Dieckmann, M. (1961) The Enzymic Synthesis of Amino Acyl Derivatives of Ribonucleic Acid. II. The Preparation of Leucyl-, Valyl-, Isoleucyl- and Methionyl Ribonucleic Acid Synthetases from *Escherichia coli*. *J. biol. Chem.*, **236**, 1735.

Brookes, P. & Lawley, P. D. (1960) The Methylation of Adenosine and Adenylic Acid. *J. chem. Soc.*, 539.

Brunngraber, E. F. (1962) A Simplified Procedure for the Preparation of " Soluble " RNA from Rat Liver. *Biochem. biophys. Res. Commun.*, **8**, 1.

Dunn, D. B. & Hall, R. H. (1970) Purines, Pyrimidines, Nucleosides and Nucleotides: Physical Constants and Spectral Properties. In *Handbook of Biochemistry*. Ed. H. A. Sober. Cleveland: Chemical Rubber Co.

Hemmens, W. F. (1964) *neo*-Guanylic Acid Produced by the Action of Acid on Ribonucleic Acid. *Biochim. biophys Acta*, **91**, 332.

Inose, M., Miyata, S. & Iwanami, Y. (1972) Methylation Patterns of tRNA from Ascites Hepatomas. *Biochim. biophys. Acta*, **259**, 96.

Iwanami, Y. & Brown, G. M. (1968) Methylated Bases of Transfer Ribonucleic Acid from HeLa and L Cells. *Arch. Biochem. Biophys.*, **124**, 472.

McCoy, T. A. & Carter, E. A. (1968) The Separation of Ribonucleic Acids on Sephadex Columns. *J. Chromat.*, **37**, 458.

Mirvish, S. S., Medalie, J., Linsell, C. A., Yousef, E. & Reyad, S. (1971) 7-Methylguanine and Other Minor Urinary Purines: Values for Normal Subjects from Israel, Gaza and Kenya and for Patients with Cancer of Various Organs or Cirrhosis of the Liver. *Cancer, N.Y.*, **27**, 736.

Randerath, K. (1971) Application of a Tritium Derivative Method to Human Brain and Brain Tumor Transfer RNA Analysis. *Cancer Res.*, **31**, 658.

Shapiro, R. & Gordon, C. N. (1964) On the Structure of Neoguanosine. *Biochem. biophys. Res. Commun.*, **17**, 160.

Srinivasan, P. R. & Borek, E. (1964) Enzymic Alteration of Nucleic Acid Structure. *Science, N.Y.*, **145**, 548.

Viale, G. L. (1971) Transfer RNA and Transfer RNA Methylase in Human Brain Tumors. *Cancer Res.*, **31**, 605.

Weissmann, B., Bromberg, P. A. & Gutman, A. B. (1957*a*) The Purine Bases of Urine. I. Separation and Identification. *J. biol. Chem.*, **224**, 407.

Weissmann, B., Bromberg, P. A. & Gutman, A. B. (1957*b*) The Purine Bases of Urine. II. Semiquantitative Estimation and Radioisotope Incorporation. *J. biol. Chem.*, **224**, 423.